# Development and Characterization of a Nutritionally Rich Spray-Dried Honey Powder

**DOI:** 10.3390/foods10010162

**Published:** 2021-01-14

**Authors:** Yogita Suhag, Gulzar Ahmad Nayik, Ioannis K. Karabagias, Vikas Nanda

**Affiliations:** 1 Department of Food Engineering and Technology, Sant Longowal Institute of Engineering & Technology (Deemed University), Longowal 148106, Distt. Sangrur (Punjab), India; er.yogita18@gmail.com (Y.S.); vik164@yahoo.co.in (V.N.); 2 Department of Food Science & Technology, Govt. Degree College, Shopian 192303, J&K, India; 3 Department of Chemistry, Laboratory of Food Chemistry, University of Ioannina, 45110 Ioannina, Greece

**Keywords:** honey powder, drying agents, antioxidant activity, glass transition temperature, XRD, FTIR

## Abstract

In the present study, the spray-dried honey powder enriched with aonla (*Emblica officinalis* Gaertn) and basil (*Ocimum sanctum*) extract was developed using drying aids—gum arabic (GA), maltodextrin (MD), and whey protein concentrate (WPC), and then characterized based on particle size distribution, colour, glass transition temperature (Tg), X-ray diffraction, and antioxidant and rheological properties. Results showed the highest Tg (86.13 °C) for WPC based honey powder, which, in turn, resulted in least stickiness as compared to GA and MD based honey powders with Tg 74.53 °C and 68.26 °C, respectively. The dried honey powder with all three carrier agents exhibited a metastable amorphous state as proved by the broader peaks of X-ray diffractograms due to the short drying time, whereas, a peak at 1637 cm^−1^, attributed to the carbonyl (C=O) stretching, established the ascorbic acid in the studied powder on account of aonla extract. The mean particle diameter significantly (*p* < 0.05) increased, following the order WPC (60.45 μm) > GA (41.24 μm) > MD (20.06 μm) as carrier agents, which were related to the higher feed viscosity. The colour parameter L* (30.74–45.78) and b* (5.82–11.64) values of the nutritionally rich honey powder were higher due to presence of polyphenols in aonla and basil extracts, which resulted in the formation of dark brown complexes. The antioxidant activity of WPC based fortified honey powder was highest (82.73%), followed by GA (78.15%) and MD (74.85%) based honey powders. A significant (*p* < 0.05) increase was found in powder recovery, solubility, and dispersibility using the drying aids in the following order: WPC < GA < MD. Furthermore, the storage modulus (G′) was higher than loss modulus (G″) in all honey powders, wherein the WPC containing powder demonstrated maximum value of G′, followed by GA and MD. Finally, the three honey powders were microbiologically stable.

## 1. Introduction

Honey is a natural sweet product having unique flavour; it is considered as a functional food because of its uncountable medical, nutritive, and antioxidative properties [1], but due to its high viscosity, crystallization, and stickiness in its natural form, it causes difficulties in mass utilization, which can be overcome by conversion of honey into powdered form [2,3]. However, the application of drying in sugar-rich foods, such as honey, is, in general, difficult, due to the presence of low-molecular weight sugars, namely glucose, fructose, and sucrose in the feed mixtures [4]. Spray-drying is a promising method of changing liquids into solids, which retains the flavour and nutrients, reduces the volume, and ensures easy handling, thus making it an ideal technology in food processing [5]. However, honey powder has higher shelf life and stability due to its multiple uses in different food formulations, such as bakery and meat products [6]. Colour, an essential organoleptic characteristic of honey powder, is influenced by several processing factors, such as inlet air temperature and carriers during spray-drying [7]. Honey powder showed lower values of glass transition temperatures (Tg) due to simple sugars of low-molecular weight, like fructose (Tg 5 °C), and glucose (Tg 31 °C) and, consequently, causes time-based structural changes, such as higher hygroscopicity, thermoplasticity, and crystallization. These structural changes may result in significant economic losses and operational issues during spray-drying [8]. At low Tg, the highly viscous (about 1012 Pa.s) components are converted to the softer, visco-elastic, and rubbery substances, having 106–108 Pa.s viscosity from the initial glassy state [9]. However, the fortification of honey powder with carrier agents of high molecular weight resolved the stickiness or caking issues by raising the Tg of amorphous food powders, causing reduction in viscosity and increase in volume and specific heat, as reported in the literature [10].The higher honey content in whey protein concentrate (WPC) based honey powder represented good physical properties, pleasant flavour, and taste as compared to gum arabic (GA) and maltodextrin (MD) [11].

On the other hand, whey protein concentrate as a novel drying aid, possesses higher emulsification, stability against oxidation, surface active nature, film-forming property, and acts as an encapsulating wall material [12]. Furthermore, the moisture level of carriers, their chemical composition and molecular weight affect the glass transition temperature [13]. The crystallinity of some principal components may be altered by spray-drying, as reflected by the amorphous state, which, in turn, can be examined using X-ray diffraction, a technique used for the state identification of powders. Both states of powder, either crystalline or amorphous, reveals the significant variations in chemical stability, flow-behaviour, hygroscopicity, particle size and shape, physicochemical properties, and water solubility [14]. Besides, the different food constituents, such as sugars, vitamin C, etc., are evaluated using Fourier transform infrared (FTIR) spectroscopy, which provides a quick, robust, and exceptional frequency precision, inexpensive qualitative analytical method, acceptable signal-to-noise (S/N) ratio, and short data acquisition period [15,16].

Aonla (*Emblica officinalis*) is indigenous to Indian sub-continent. Globally, India is leading in production of this crop. It is also found naturally in Sri Lanka, Cuba, Puerto Rico, USA (Hawaii and Florida), Iran, Iraq, Pakistan, China, Malaysia, Bhutan, Thailand, Vietnam, Philippines, Trinidad, Panama, and Japan. Aonla is a good source of vitamin C, which possesses high antioxidant, anti-inflammatory, and hepato-protective activity [17]. The aonla fruits are used in management of diseases since ancient times [18].

*Ocimum sanctum* (*Basil*) is commercially produced in India as a most sacred herb. It possesses antioxidant, antiviral, antifungal, anticancer, anti-stress, antidiabetic, anti-inflammatory and antifertility properties. Basil is generally employed in the treatment of various diseases, such as arthritis, chronic fever, eye and heart disease, etc. [19].

Tonon et al. [20] reported the reduction in total phenolic content (TPC) and antioxidant potential of spray-dried acai powder due to higher inlet temperatures. Similarly, Chaikham and Prangthip [21] reported an extensive decrease in TPC and antioxidant activity of honey under heating (at 100 °C for 5 min). Therefore, the present study was conducted with the objective to develop a nutritionally rich honey powder enriched with functional aonla and basil extracts using different carriers (GA, MD, and WPC) for improving the antioxidant potential, TPC, and ascorbic acid content. In addition, the fortified powder was characterized on the basis of Differential Scanning Calorimeter (DSC), X-ray diffraction, FTIR, particle size, and colour analyses.

## 2. Materials and Methods

### 2.1. Materials

*Helianthus annuus* honey was confirmed for its botanical origin using the pollen amount (≥45%), as per the frequency ratio of each pollen in honey [22]. A standard classification involving predominant pollen (>45% of counted pollen grains), secondary pollen (16–45%), important minor pollen (3–15%), and minor pollen (<3%) was employed to study the pollen frequency distribution.

Gallic acid, gum arabic, maltodextrin (DE-20), Folin-Ciocalteu reagent, and sodium carbonate were procured from LobaChemie Pvt. Ltd., Mumbai (India), whereas acetone,2,2-diphenyl picrylhydrazyl (DPPH), and HPLC grade methanol were purchased from Ranbaxy, New Delhi (India). The whey proteins concentrate (70% protein) was procured from Mahaan Proteins Ltd., New Delhi (India) and aonla (Neelam variety) and basil leaves (holy basil) were procured from Punjab Agriculture University, Ludhiana (India), and local farmers, respectively.

### 2.2. Sample Preparation and Spray Drying

Due to high viscosity, the honey-water (1:3.5) mixture was prepared to minimize the clogging and three drying aids: gum arabic (45% *w/w* of honey), maltodextrin (DE-20) (50% *w/w* of honey), and whey protein concentrate (35% *w/w* of honey), were added to this mixture. The feed mixture was prepared by incorporating the aonla (*Emblica officinalis* Gaertn) (8%) and basil (*Ocimum sanctum*) (6%) after complete dissolution of the carrier agent [11]. The spray-drying of honey was performed in a tall type laboratory scale spray- dryer (S.M. Scientech, Calcutta, India) having concurrent air flow. The peristaltic pump was employed to feed the honey blend, while pump-rotation speed controlled the flow rate of feed. During the experiment runs, the process variables, such as blower speed, feed rate, inlet air temperature, and outlet temperature were fixed to 2000 rpm, 0.11 mL/s, 170 °C, and 90 °C, respectively. On the completion of drying process, an insulated glass jar connected to cyclone-end was employed to collect the powder. The fortified powder was then transferred to polyethylene bags and stored in the desiccators until further analysis.

#### 2.2.1. Viscosity

Feed mixture viscosity (millipascal seconds) was analysed using Brookfield Viscometer Engineering Laboratories Inc. (Stoughton, MA, USA). The viscosity was measured in triplicate using a constant volume (16 mL) and varying shear rates (0 to 300 s^−1^) at 25 °C.

#### 2.2.2. Water Activity

A water activity meter (AquaLab 3TE Series, Decagon, Pullman, WA, USA) was used to determine the water activity of the fortified powder samples. The measurements were carried out immediately after the drying process.

#### 2.2.3. Powder Recovery

It was calculated using the following mathematical formula:

(1) $$\mathrm{Powder}\;\mathrm{Recovery}\;(\%)=\frac{\mathrm{Weight}\;\mathrm{of}\;\mathrm{developed}\;\mathrm{powder}\;\left(\mathrm{dry}\;\mathrm{basis}\right)\;}{\mathrm{Total}\;\mathrm{solid}\;\mathrm{conent}\;\mathrm{in}\;\mathrm{feed}}\times 100$$

#### 2.2.4. Bulk Density

A known weight of powder samples was poured gently into a graduated empty measuring cylinder, then tapped 20–25 times, and the volume was then recorded [23].

#### 2.2.5. Absolute Density

A pycnometer was used to estimate the absolute density (ρ_abs_) wherein the ethanol (99%) was an immiscible liquid [2].

#### 2.2.6. Porosity

Porosity was determined from the link between bulk and absolute density of powder samples, as shown below [14]:

(2) $$\mathrm{Porosity}=1-\frac{\mathrm{Bulk}\;\mathrm{density}}{\mathrm{Absolute}\;\mathrm{density}}$$

#### 2.2.7. Dispersibility

The dispersibility of the fortified powders was estimated according to the methodology of Jaya and Das [24]. Briefly, 100 g water was added to 30 g honey powder in a beaker and stirred at 50 °C to complete 25 back and forth movements through the beaker, covering its entire diameter for 15 s. This mixture was then passed through the sieve (210 μ) and the moisture content of filtered portion was determined by heating the sample at 105 °C until constant weight to calculate the dry matter. The dispersibility was then estimated using the following formula:

Dispersibility = (W + a × Sp)/(a + Sj)(3)

where, “W” refers to the weight of water used in reconstitution, “a” is the amount of powder used (g), “Sp” refers to total solids (%) found in powder samples, and “Sj” is dry matter (%) obtained by passing the honey powders mixture through the sieve.

#### 2.2.8. Solubility

Solubility was determined using the procedure followed by Cano-Chauca et al. [25]. Moreover, 1 g of powder was added to blender jar, containing 100 mL of distilled water. It was operated for 5 min at 15,000 rpm and the obtained mixture was then centrifuged at 3000× *g* for 5 min. Afterwards, a volume of 25 mL of the supernatant was poured into the pre-weighed Petri plates for drying at 105 °C for 5 h, and the weight difference was used to calculate the solubility (%).

#### 2.2.9. Hygroscopicity

Hygroscopicity was determined using the method of Cai and Corke [26], wherein 1 g of sample was kept in a container filled with a saturated NaCl solution (75% RH) for 1 week at 25 °C. The sample was then weighed and the adsorbed moisture (g per 100 g dry solids) was used to indicate the hygroscopicity.

#### 2.2.10. Total Phenolic Content (TPC), Antioxidant Activity (AOA), and Vitamin C Content

The honey powder samples were analysed according to the methods of Liu et al. [27] and Luo et al. [28] for TPC and AOA, respectively, whereas vitamin C content was also determined according to method adapted by Luo et al. [28].

#### 2.2.11. Particle Size Distribution

A laser light diffraction instrument (Shimadzu SALD-2300, Kyoto, Japan) was used to determine the particles size distribution of the fortified powder samples, wherein the isopropanol (1.37 refractive index) was employed as a dispersion medium to suspend the small amount of powder. Magnetic agitation was used for uniform dissolution of powder and the particle size analysis was performed using a particle size analyser. Each experiment was run in triplicate and the median diameter, was estimated by the instrument, and referred to the particle size of powder, which showed the particle diameter at 50% of the normalized particle amount.

#### 2.2.12. Colour

The colour values were measured with a colour spectrophotometer (CM-3600d, Konica, Minolta, NJ, USA) by measuring of L*, a*, and b* values. L* represented the lightness ranging from black to white, whereas a* and b* values demonstrated the red–green and blue–yellow components. The *Chroma* (C*) and Hue (*H*) were calculated using the following Equations (4) and (5):

C* = (a*^2^ + b*^2^) ½ (4)

*H* = tan^−1^(b*/a*) (5)

Further, the total colour difference (Δ*E*) was determined using the following mathematical expression as:

(6) $$\Delta E=\sqrt{{\left(L_{0}{}^{*}-L^{*}\right)}^{2}+\;{\left(a_{0}{}^{*}-a^{*}\right)}^{2}{}^{*}+\;{\left(b_{0}{}^{*}-b^{*}\right)}^{2}}$$

where, *L*_0_*, *a*_0_* and *b*_0_* are the colour values of samples while L*, a* and b* represented the respective values when aonla (8%) and basil (6%) extracts were used in the preparation of powder.

#### 2.2.13. Glass Transition Temperature (Tg)

A Differential Scanning Calorimeter (DSC, Model-821, Mettler-Toledo, Greifensee, Switzerland) was employed to determine the glass transition temperature, Tg of powder. Nitrogen (25 mL/min) was used as purge gas whereas indium and zinc (Perkin-Elmer standards) were used to calibrate the temperature and heat flow, respectively. The powder samples were brought to set temperature (25 °C) using quick-cooling to reach the equilibrium. DSC aluminium (Al) pans (50 μL) were hermetically sealed to scan the 4 mg of sample while an empty Al pan was employed as the reference sample. The scanning range varied from −40 to 150 °C following the heating at a rate of 10 °C/min. Further, the STARe software (Version 8.1 Mettler-Toledo, Greifensee, Switzerland) were used to calculate the midpoint values.

#### 2.2.14. X-ray Diffraction (XRD)

The XRD patterns were obtained using Cu based anode X-ray tube of an analytical X-ray Diffractometer (X’Pert PRO, Panalytical, Almelo, Netherlands). The powder sample was tightly pressed using glass slide in an aluminium holder. The measurements were performed using the diffraction angle ranging from 4 to 40° (2θ) with a scanning rate of 4°/min at 30 mA and 40 kV.

#### 2.2.15. FTIR Spectroscopy

The FTIR spectrum of honey powder samples was obtained with the use of Agilent Cary 660 FTIR spectrophotometer (Agilent Technologies, CA, USA) following the scanning range of 4000–400 cm^−1^ in transmittance mode at ambient conditions.

#### 2.2.16. Dynamic Rheological Measurement

The rheological properties of powder samples were determined using Modular Compact Rheometer (MCR-102, M/s. Anton Paar, Graz, Austria), which contained parallel-plate system (50 mm dia) following 0.5 mm gap at a temperature ranging from 0 to 40 °C at an interval of 10 °C. The frequency sweeps varied from 0.1 to 100 rad/sat 3% strain and the dynamic rheological data (G′ and G″) was derived using a Rheoplus data analysis software (32 V3.40).

#### 2.2.17. Microbiological Analysis

The total microbial count of the fortified powders was determined according to the Indian Standard Institution (ISI) method (IS 5402:2012).

#### 2.2.18. Sensory Evaluation

Sensory evaluation was carried out to determine the quality of the fortified honey powders using the human senses. During the sensory analysis, eight panellists were supplied with a glass of water and asked to rinse their mouth and discard water between the analyses of the different samples. The panellists shared their feelings about samples by providing scores as per the 9-point hedonic scale (Table 1) to each of the following properties: colour, mouthfeel, texture, sweetness, sourness, taste, and overall acceptability.

Panellists represented both males and females whose ages varied between 25 and 48. All panellists were trained and selected on the basis of their interests, motivations, attitudes to foods, health, and knowledge of food powders.

### 2.3. Statistical Analysis

The tests were performed in triplicate; five of each sample and experimental results were expressed as mean values ± standard deviation (SD) for each sample. An analysis of variance (ANOVA) was conducted and Duncan’s Multiple Range Test (DMRT) was employed to determine the least significant difference at *p* < 0.05using Statistica software.v.12 (StatSoft India Pvt. Ltd., New Delhi, India).

## 3. Results and Discussion

### 3.1. Viscosity

Viscosity of honey and all of the prepared feed mixtures in the present study are reported in Table 2.

Honey has been reported to behave as a Newtonian fluid [29], and all of the feed mixtures also followed a Newtonian behaviour, despite the difference in the carrier agents. Our results are in good agreement with those reported by Samborksa et al. [3]. The estimated viscosity of the prepared feed mixture using maltodextrin (27.32 mPa s) and gum arabic (35.56 mPa s) as carrier agents were higher when compared with that of the feed mixture produced with WPC (22.12 mPa s). Similarly, the feed solution showed an increase in maltodextrin and gum arabic total solid ratio, as reported by Samborksa et al. [3].

### 3.2. Water Activity

The water activity determines the free and available moisture attributed to any kind of biochemical reactions and is considered as a key parameter for assessing the microbiological stability of food products. In the present study, the fortified powders with MD, GA, and WPC showed a water activity of 0.289, 0.242, and 0.195, respectively, which were less than 0.3 (Table 3), demonstrating that the developed powders were microbiologically quite stable.

Usually, the higher value of water activity shows the higher concentration of free moisture, and thus, shelf-life may be reduced due to biochemical reactions; however, this is not relevant to the results of the present study. The data about water activity were almost similar to those reported by Tonon et al. [20] and Daza et al. [30], while drying the cagaita fruit and acai, respectively. The water activity of the fortified honey powder with WPC was the lowest among the other developed powders, on account of its amorphous nature, which possess high water holding potential [12].

### 3.3. Powder Recovery

The efficiency of powder collection shows the powder recovery of the drying process. A spray dryer loses the powder because the sprayed droplets of the powder is adhered to the drying chamber, as well as in the cyclones, while collecting fine particles. As shown in Table 3, the powder recovery of MD, GA, and WPC based honey powders varied from 53.44 to 65.10%. In the present study, the powder recovery (%) increased on adding three drying aids, a phenomenon that may be attributed to the difference at molecular levels among MD, GA, and WPC, which usually have higher values of Tg due to their larger molecules. The higher powder recovery obtained for WPC as carrier agent might be due to the proteins and their surface activity; therefore, forming an interface of feed mixture, which then generates a film after/during drying. This phenomenon reduces the association between chamber wall and droplets resulting in a reduced stickiness. The data of the present study are in accordance with those of Fang and Bhandari [31], concerning the drying of bayberry juice, using various amounts of whey protein isolate, where the powder recovery ranged from 45.6 to 56.2%.

### 3.4. Absolute Density

Normally, absolute density is associated with the actual solid density, which excludes the inter-particle voids, whereas the bulk density considers such voids during analysis. In the present study, the absolute density increased with the amounts of drying aids and ranged between 1.52 g/mL and 1.80 g/mL (Table 3). The MD based powder showed a slightly higher absolute density due higher inter-particle spaces followed by GA based honey powder, which has relatively lesser inter-particle spaces. The sample fortified with maltodextrin showed a significant increment in absolute density, which was also reported by Tonon et al. [20] and Ferrari et al. [32].

### 3.5. Porosity

Porosity is an essential attribute of microcapsules where the encapsulated substance is extremely vulnerable to oxidation. Porosity is being linked with bulk and absolute density that measures the fractional volume occupied by air. The fortified powder with WPC was expected to possess the highest porosity in comparison to the other carrier agents, since it forms a powder with a greater particle size due to the protein content, as shown by other authors [33]. Nevertheless, we did not detect any significant difference (Table 3).

### 3.6. Solubility

Solubility is an important criterion to determine the behaviour of powder in an aqueous phase. The food powders, when examined in an aqueous phase, had a characteristic solubility. The data related to the solubility of fortified honey powders using three drying aids are shown in Table 3. The solubility of the fortified powders increased with the concentration of carriers due to their highly water-soluble nature. As shown, the WPC containing honey powder had the maximum solubility attributed to the higher water holding potential of proteins. Our results were similar to gum arabic and inulin based spray-dried cagaita fruit extracts [30].

### 3.7. Dispersibility

The ability to achieve a uniform distribution in solution is referred to as dispersibility. Table 3 shows that dispersibility of honey powders ranged from 67.55% to 81.47% and increased with the addition of carrier agents due to their emulsifying properties, which in turn may be attributed to the amphiphilic molecular structures of aids. The lumps and agglomeration are less present in water when powder dispersibility is high [34]. A significant (*p <* 0.05) effect of drying aids on dispersibility is shown in Table 3. The inter-particle and particle–liquid interactions influence the technical attributes of flowability, as well as dispersibility of spray-dried microparticles [35].

### 3.8. Hygroscopicity

The hygroscopic nature of the fortified powders is linked with their higher sugar content. The lower hygroscopicity of WPC (23.14%) as compared to GA (25.43%) and MD (26.39%) (Table 3), which could be attributed to higher molecular weight of drying aids. The water was adsorbed in lower amounts by MD and GA due to their quick water adsorption, which then alters the equilibrium between hydrophilic/hydrophobic sites of powder. The hygroscopicity of WPC was the lowest among all carriers (*p* < 0.05) due to the exceptional surface activity and film-forming traits of proteins, thus raising the Tg. Present results are in good agreement with those reported by Wang et al. [36] while developing a spray-dried soya sauce powder.

### 3.9. Particle Size

The determination of particle size is essential to design and monitor the food processing operations, such as handling, storage, and quality control. The fortified powders revealed the highest mean diameter (D_50_) when drying aids were present (Table 3). MD, GA, and WPC had D_50_ values of 20.06 μm, 41.24 μm, and 60.45 μm, respectively, which contributed to bulk density. Thus, WPC based honey powder had low bulk density while MD based honey powder possessed high bulk density in the present investigation. A similar behaviour was observed with spray-dried black raspberry juice powder [37].

During drying, bigger particles were obtained because WPC concentration increased exponentially the feed viscosity, leading to the formation of larger droplets during atomization. Similar effects were observed by Du et al. [38] and Muzaffar and Kumar [39], while drying the persimmon pulp and tamarind pulp (using MD and soy protein isolate), respectively.

Large particles hold more space, leading to an easier water penetration, whereas the smaller particles are less porous, thus making the water penetration quite tough, and thereby leading to reduced reconstitution properties. Furthermore, the particle size distribution may affect the mixing behaviour of constituents, density, and individuality of a blend and flow out of storage bins, whereas the particles that are smaller in size are settled at the bottom, and the bigger particles remained at the top surface.

### 3.10. Colour

Table 4 shows the colour of the nutritionally rich honey powder, as affected by drying temperature and different carriers (MD, GA, and WPC).

The fortified powder with WPC showed significantly (*p* < 0.05) lower values of lightness (L*) as compared to the other powder samples due to the inherent yellowish colour of the WPC and lower addition rate than MD and GA. Present results are in good agreement with those reported by Bhusari et al. [40] during the development of tamarind pulp powder using spray-drying. Furthermore, higher a* values were obtained for honey powder fortified with WPC and GA, compared to the MD based powder, possibly due to the Maillard reaction between reducing sugars and amino acid of protein portions found in GA and WPC. Honey powder with WPC showed higher b* value than honey powder made with GA and MD. Table 4 shows that a significant difference among samples produced with different drying aids was observed, with respect to colour parameters.

Chroma* and Hue (°) describe the saturation and perception of colour, respectively. The use of WPC as a carrier agent significantly increased the Chroma* values (11.84) and decreased the hue (79.41°), resulting in the generation of a golden yellow powder. These results might be associated with an inherent colour of WPC owed to specific pigments, leading to the hypothesis that may act as a good encapsulant with high antioxidant activity. Similar findings were reported by Ferrari et al. [32] who worked with blackberry pulp powder.

The colour value of Δ*E* was the highest for the fortified powders with aonla and basil extracts, along with WPC (10.37), MD (10.06), and GA (8.76), than the powders without these extracts. The reason behind this observation may be the encapsulation of these extracts inside the WPC, which might have increased the TPC content by synthesizing brown-coloured complexes, due to the interaction with amino acids and proteins. However, no earlier studies have been carried out relevant to the present findings.

### 3.11. Total Phenolic Content (TPC), Antioxidant Activity (AOA), and Vitamin C Content

The values of TPC, AOA, and ascorbic acid of the fortified powders with MD, GA, and WPC, along with aonla and basil extracts, are given in Table 5. The honey powder containing aonla and basil extracts showed an increase in TPC, AOA, and ascorbic acid content. Higher retention of polyphenolic compounds and vitamin C content, which possess antioxidant potential, was achieved with WPC, followed by gum arabic and maltodextrin. Such retention might be possible because WPC protected the compounds from oxidation and showed exceptional emulsification properties. Flores et al. [41] reported similar findings in a study concerning the antioxidant potential of spray-dried blueberry pomace extracts.

### 3.12. Glass Transition Temperature (Tg)

**Figure 1:**
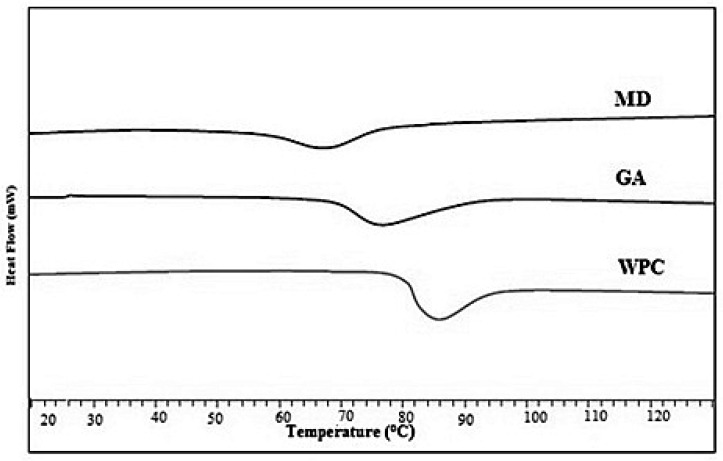
Glass transition temperatures of spray-dried nutritionally rich honey powders fortified with maltodextrin (MD), gum arabic (GA), and whey protein concentrate (WPC) carrier agents.

Various studies have shown that surface stickiness of an amorphous powder is closely linked with glass transition temperature. The Tg value of liquid honey is very low (42.95 °C) due to the presence of simple sugars (of lower molecular weight), including glucose and fructose, and, thus, plasticization effect of water results in the stickiness of honey during spray drying. The Tg values of the fortified powders with MD, GA, and WPC ranged significantly (*p* < 0.05), and were 68.26 °C, 74.53 °C, and 86.13 °C, respectively (Figure 1).

An increase in Tg value refers to the improved stability of honey powder. In the current study, the honey powder containing MD showed the lowest Tg value than powder samples fortified with GA and WPC. The higher levels of maltodextrin increased hydrophilic groups and shorter chains, resulting in low Tg. Tonon et al. [20] reported a similar behaviour of spray-dried acai juice using different drying aids. The WPC based honey powder contained the droplets with active surface layer in which the Tg value was greater as compared to the respective bulk Tg values, which led to the decrease in particle–wall stickiness. Therefore, the WPC can be an ideal carrier for thermally sensitive products while drying. Fang and Bhandari [31] reported an increase in Tg values up to 16 °C for spray-dried bayberry powder when whey protein isolate (0.5–10%) was used as a drying aid. However, all three drying aids were effective to minimize the stickiness issues due to the increase in Tg values while spray-drying of honey.

### 3.13. XRD Analysis

**Figure 2:**
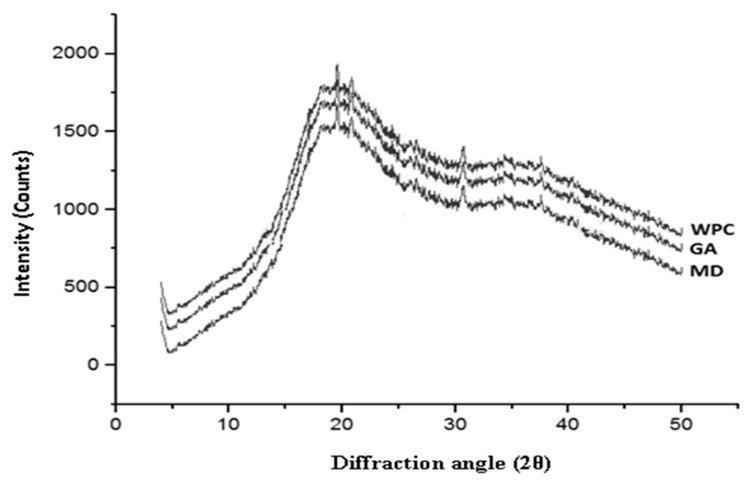
XRD pattern of nutritionally rich honey powders fortified with different carrier agents.

XRD determines the crystalline or amorphous state of dried powders. Usually, several sharp peaks are present in highly-ordered state in a crystalline material while disordered and dispersed bands are displayed in the molecules of amorphous products [14]. In the current study, the diffractograms of the fortified powders with WPC, GA, and MD showed amorphous characteristics due to the short contact time during spray-drying (Figure 2).

Further, the insufficient crystallization time of simple or more complex sugars (glucose, fructose, and sucrose) might induce the variations in crystallinity of major compounds, resulting in an amorphous metastable state of honey powder. Similar findings were reported by Wang and Zhou [36]. The intensity of diffraction peak of WPC based honey powder was the maximum, compared to the intensities of honey powder fortified with MD and GA. This may be due to the fact that the honey powder fortified with WPC might have partially crystallized, due to the longer drying time [42]. Higher glass transition temperature and presence of metastable amorphous peaks of honey powder with these three carriers confirmed that there was no significant change in the hygroscopicity of honey powder.

### 3.14. FTIR Spectroscopy

**Figure 3:**
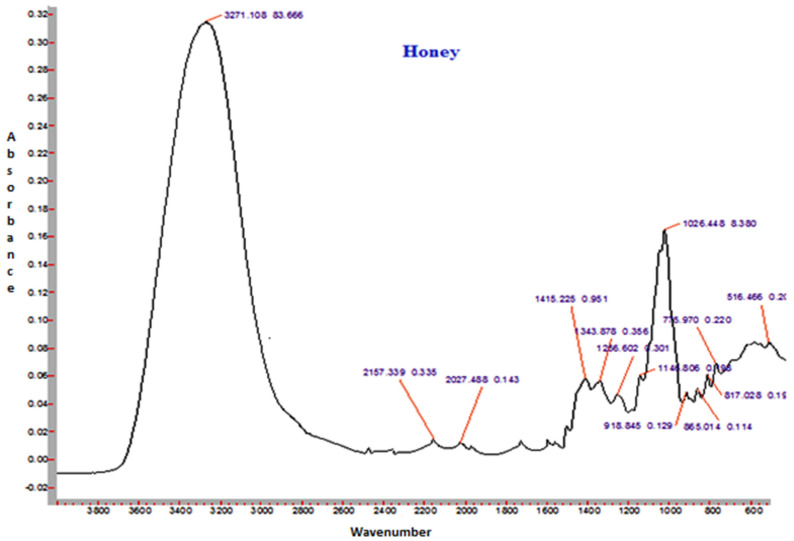
Representative FTIR spectrum of sunflower honey.

**Figure 4:**
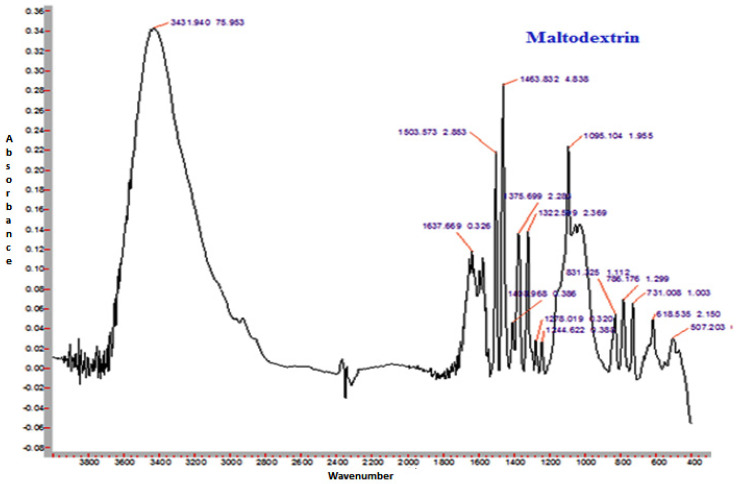
Representative FTIR spectrum of spray-dried nutritionally rich honey powder fortified with maltodextrin (MD).

**Figure 5:**
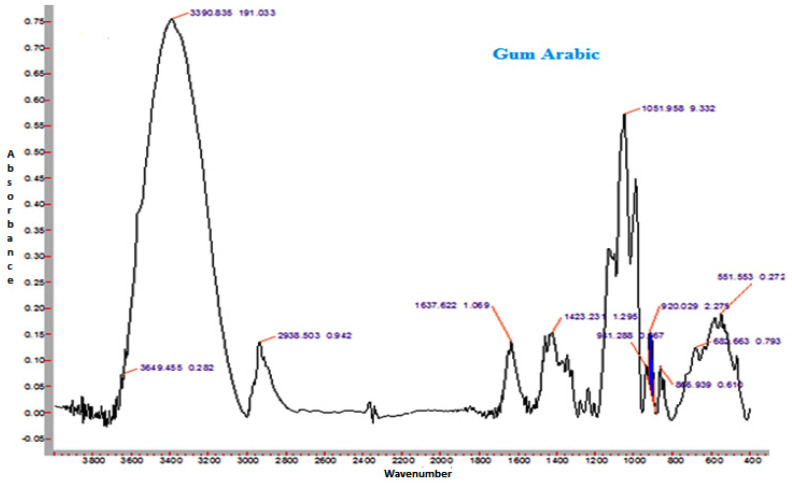
Representative FTIR spectrum of spray-dried nutritionally rich honey powder fortified with gum arabic (GA).

**Figure 6:**
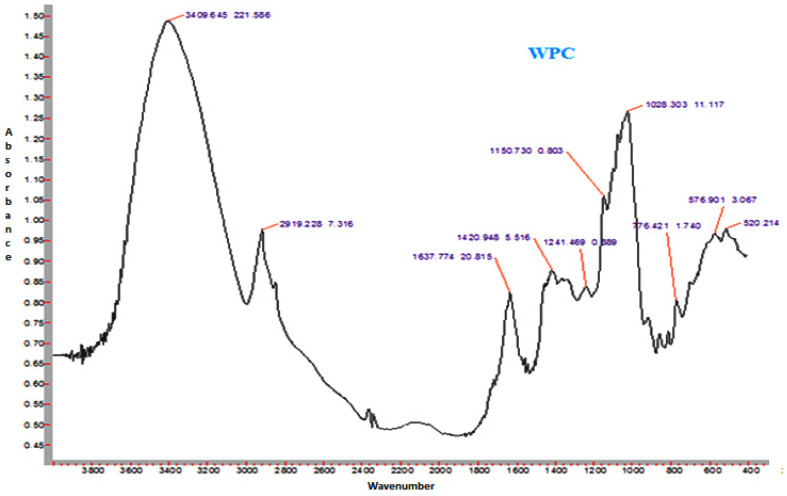
Representative FTIR spectrum of spray-dried nutritionally rich honey powder fortified with whey protein concentrate (WPC).

The FTIR spectra of sunflower honey and honey powder samples fortified with MD, GA, and WPC in the present study are shown in Figure 3, Figure 4, Figure 5 and Figure 6, respectively. It was observed that the spectral region between 1000 and 1500 cm^−1^ of honey and honey powders would cover most of the characteristic absorption bands relevant to major sugars.

The spectra of honey powder produced with whey protein concentrate shows bands of fructose and glucose at 1028 and 1420 cm^−1^, which corresponds to the C-O stretching in -COH group and C-C stretching in carbohydrates. Similar findings were reported by Nayik et al. [29] for various mono-floral Indian honey samples. In the same figures, band at 2938 cm^−1^ corresponds to N-H stretching due to the addition of whey protein concentrate as carrier, whereas the broad band at 3409 cm^−1^ was due to the hydroxyl (O-H) stretching vibration. The variation of FTIR spectra of MD and GA based honey powder is presented in Figure 3. The key peak transmittance bands of fructose were 1095 cm^−1^ and 1051 cm^−1^, which are usually associated with C-O bending and C-OH stretching of honey powder fortified with maltodextrin and gum arabic, respectively. The absorbance key peak of glucose at 1322 cm^−1^ and 1344 cm^−1^ is ascribed to O-H bending of MD and GA based honey powders, respectively. Similar bands of glucose and fructose were reported by Wang et al. [43] in honey samples of different geographical regions. The honey powder fortified with maltodextrin and gum arabic dominated the water bands at 3431 cm^−1^ and 3390 cm^−1^ (O-H stretching), respectively, and may be associated with the synthesis of glycosidic linkage of sugars.

Some additional sharp peaks at 1637 cm^−1^ ascribing to carbonyl group (C=O) stretching and 576 cm^−1^ contributed to C-H out of plane band for H-C=C group, and were observed in the spectra of honey powders fortified with GA and WPC. These are probably attributed to the functional groups of vitamin C. This might be due to the encapsulation of aonla (*Emblica officinalis* Gaertn) extract, which is the richest source of ascorbic acid, with WPC in honey powder. The band at 2938 cm^−1^ and 2919 cm^−1^ corresponded to N-H stretching due to the addition of gum Arabic and WPC, respectively.

### 3.15. Rheological Characteristics of Optimized Nutritionally Rich Honey Powders

**Figure 7:**
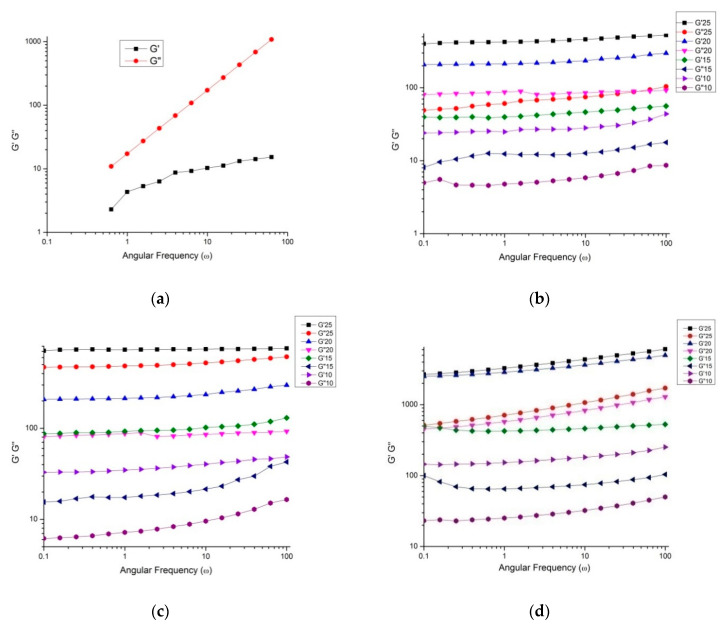
Storage modulus (G′) and loss modulus (G″) as a function of angular frequency (**ω**) for (**a**) honey and nutritionally rich honey powders developed using (**b**) MD, (**c**) gum arabic and (**d**) WPC respectively, at different concentrations.

The dynamic frequency sweep rheogram of loss or viscous (G″) and storage or elastic modulus (G′) is shown in the context of angular frequency (1 to 100 rad/s) for sunflower honey and honey powders at different concentrations (10%, 15%, 20%, and 25%) at 20 °C (Figure 7a–d).

Present results exhibited the increase in both magnitudes G′ and G″ with the increase in angular frequency throughout the frequency span. However, the crossover phenomenon did not occur with an increase in frequency. G″ magnitude, which depends on frequency, was significantly higher compared to the G′ across the entire frequency, revealing that sunflower honey had less elastic nature than being viscous (G″ ≫ G′). Several studies reported similar rheological characteristics of numerous honey types from different regions [44]. More specifically, for honey powders, G′ was greater than G″ and maximum increase G′ was obtained for honey powder fortified with WPC, followed by GA and MD. This trend may be attributed to an increase in elasticity due to macromolecular network formation, in which the molecule is consisted of long chain and strongly solvated particles, thus generating a concentrated network. This behaviour has been observed for aloe-vera mucilage during spray-drying [45]. Therefore, honey presented Newtonian behaviour, and it was converted into non-Newtonian behaviour for all of the honey powders studied herein, due to addition of carrier agents during spray-drying. The Newtonian behaviour was shown by sunflower honey, which is not dependent on strain, and such honeys usually crystallizes quickly on account of the higher sugar constituents when examined for rheological properties.

**Figure 8:**
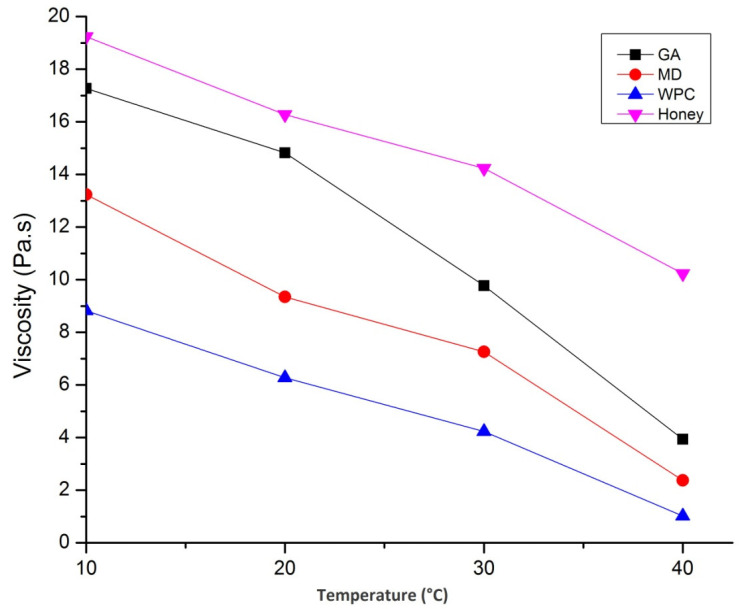
Effect of temperature on viscosity of honey and nutritionally rich honey powders fortified with maltodextrin (MD), gum arabic (GA), and whey protein concentrate (WPC).

The temperature-dependent viscous nature of sunflower honey and fortified powders is shown in Figure 8.

The viscosity of sunflower honey decreased with increase in temperature due to the reduction in mean inter-molecular forces, but there was also an increase in kinetic energy, thus enhancing the mobility of molecules. Similar findings have also been reported by Nayik et al. [29]. Besides, the viscosity of GA containing honey powder was highest followed by MD and WPC. The addition of different carrier agents resulted in an increase in viscosity due to the presence of molecules, such as polysaccharides and proteins, and, thus, the resulting network was stabilized by the intermolecular association of hydrogen bonding, van der Waal interactions, and electrostatic forces. The complicated structure without inter-molecular cross-linkages were responsible for the high viscosity in spray-dried catfish roe protein emulsion system. The increase in temperature, up to 40 °C, increased the viscosity of all three honey powders; however, temperature beyond that value decreased the viscosity. During heating, the interactions between honey and carrier agents were slightly disrupted, resulting in a slight increase in viscosity. However, viscosity decreases due to elevated temperature and this phenomenon may result in the loss of the polymeric networks present in the honey powders. Similar findings have also been reported by Kiran and Rao [46].

### 3.16. Microbiological Analysis

The dried powders were examined for the fungal and bacterial growth to determine the microbial load and consumer safety of the product. The microbial count of the fortified honey powders using drying aids ranged between 1.2 × 10^4^ cfu/g to 3.5 × 10^4^ cfu/g (Table 3). Yeast and moulds grow at a_w_ above 0.6; however, in the present study a_w_ was found below 0.3, which led us to feel confident about the microbial stability of all three honey powders. The water mobility may be reduced due to the transformation of matrix to glassy state from the rubbery state, which prevents the metabolic activity of bacterial cells, resulting thus, in the prolonged shelf-life of powders. Present findings are consistent to those of Yang et al. [16], where the thermal degradation of microorganisms reduced the microbial load at high inlet air temperature (170 °C) and outlet temperature (90 °C). Furthermore, the statistical analysis showed a significant effect (*p* < 0.05) of all drying aids on the microbial count of honey powders.

### 3.17. Sensory Evaluation

**Figure 9:**
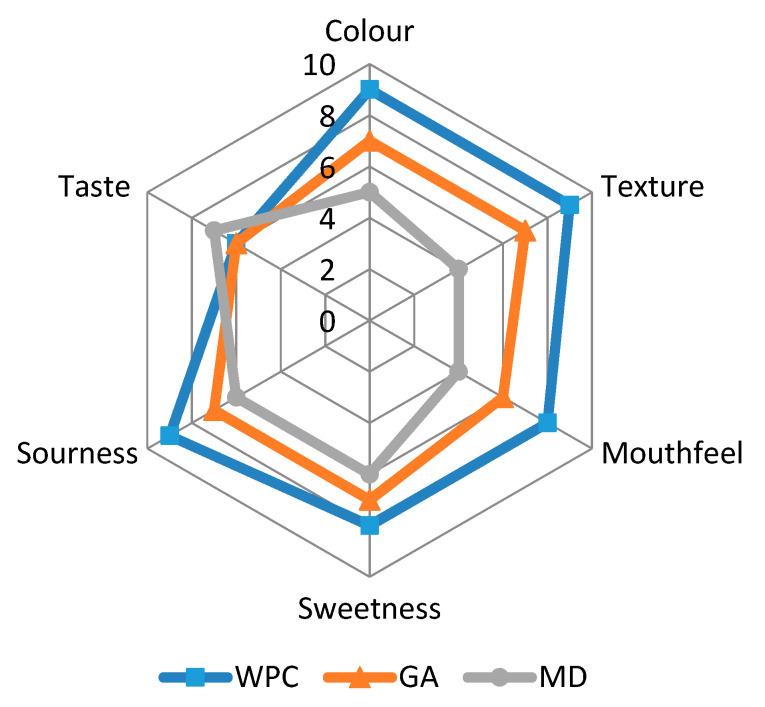
Sensory evaluation of spray-dried nutritionally rich honey powders fortified with maltodextrin (MD), gum arabic (GA), and whey protein concentrate (WPC) carrier agents.

Sensory analysis consists of numerous robust, complex, and subtle devices for measuring the human response against food products. The fortified honey powders were evaluated for sensory properties and their profiling graph is presented in Figure 9.

The WPC containing powder showed the higher scores concerning colour, texture, mouthfeel, sweetness, sourness, and overall acceptability, as compared to the other two carriers. The texture of honey powders is contributed by free-flowing nature of drying aids. The panellists experienced better texture of honey powders fortified with WPC, probably because of the reduced surface area of particle per unit mass for inter-particle bonding and mechanical inter-locking. Sweetness, as well as sourness of honey powder fortified with WPC was highly observed, followed by GA and MD. The reason behind the higher sweetness and sourness of honey powder fortified with WPC was attributed to the better encapsulation of sugars (present in honey) and vitamin C content (due to the added aonla extract) by WPC. The sourness may be derived from the synthesis of insoluble aggregates on account of reactions between tannins and salivary proteins, which result in the dried oral surface while ingesting the aonla or the tannin-concentrated foods [47]. Mouthfeel of WPC containing honey powder was marked best by panellists as this powder presented low stickiness compared to the powders fortified with GA and MD. The taste of honey powder fortified with WPC was not acceptable, due to the off-flavour of WPC; however, the overall acceptability of the honey powder was good, in agreement with the results of Osorio et al. [6]. The overall acceptability for GA and MD based honey powders was also up to the mark.

## 4. Conclusions

The honey powders nutritionally enriched with aonla and basil extracts exhibited an increased amount of TPC and ascorbic acid content. WPC was found as the most effective among the three carrier agents used, on the basis of powder recovery, solubility, particle size, and colour. The highest glass transition temperatures, as well as metastable amorphous state of WPC containing honey powder, resulted in a decreased hygroscopicity, and encapsulation of ascorbic acid in powder from aonla extract was documented by the FTIR spectra. A non-Newtonian behaviour was shown for the fortified powders, which had higher values of G’ compared to G”, thus assuring the predominance of the solid nature of powders. The best sensory attributes, including sweetness, texture, and overall acceptability of honey powders, were obtained for the honey powder fortified with WPC.

## Figures and Tables

**Table 1:** Score card for sensory analysis of honey powders fortified with MD, GA and WPC as carrier agents.

Rating	Scale
Dislike extremely	1
Dislike very much	2
Dislike moderately	3
Dislike slightly	4
Neither likes nor dislike	5
Like slightly	6
Like moderately	7
Like very much	8
Like extremely	9

MD—maltodextrin, GA—gum arabic, WPC—whey protein concentrate.

**Table 2:** Viscosity of honey and feed mixtures fortified with maltodextrin (MD), gum arabic (GA), and whey protein concentrate (WPC) carrier agents with aonla and basil extracts.

Samples	Viscosity (m Pa s)
Honey	3.48 ± 0.45 ^d^
MD	27.32 ± 0.23 ^b^
GA	35.56 ± 0.75 ^a^
WPC	22.12 ± 0.39 ^c^

Results are expressed as mean values ± standard deviations. Means in a column with same superscripts (a, b, c, d) are not significantly different (*p* < 0.05).

**Table 3:** Physicochemical and microbiological analysis parameters of spray dried nutritionally rich honey powders fortified with maltodextrin (MD), gum arabic (GA), and whey protein concentrate (WPC) carrier agents.

Properties	MD	GA	WPC
Water activity (a_w_)	0.289 ± 0.05 ^a^	0.242 ± 0.04 ^b^	0.195 ± 0.06 ^c^
Powder recovery (%)	53.44 ± 1.5 ^c^	59.39 ± 0.9 ^b^	65.10 ± 1.7 ^a^
Moisture content (%)	3.58 ± 0.1 ^c^	4.36 ± 0.1 ^b^	4.92 ± 0.2 ^a^
Bulk density (g/mL)	0.58 ± 0.07 ^a^	0.56 ± 0.08 ^b^	0.50 ± 0.05 ^c^
Absolute density (g/mL)	1.80 ± 0.01 ^a^	1.64 ± 0.02 ^b^	1.52 ± 0.12 ^c^
Porosity (%)	0.684 ± 0.030 ^a^	0.678 ± 0.040 ^a^	0.688 ± 0.050 ^a^
Solubility (%)	68.14 ± 0.77 ^c^	75.39 ± 0.70 ^b^	82.17 ± 0.84 ^a^
Dispersibility (%)	67.55 ± 0.04 ^c^	74.93 ± 0.06 ^b^	81.47 ± 0.08 ^a^
Hygroscopicity (%)	26.39 ± 0.4 ^a^	25.43 ± 0.3 ^b^	23.14 ± 0.2 ^c^
Particle Size (μm)	20.06 ± 0.04 ^c^	41.24 ± 0.27 ^b^	60.45 ± 0.11 ^a^
Spore Counts (cfu/g)	3.5 × 10.0^4 a^	2.4 × 10.0^4 b^	1.2 × 10.0^4 c^

Results are expressed as mean values ± standard deviations. Means in a row with same superscripts (a, b, c) are not significantly different (*p* < 0.05) using Duncan’s multiple range test.MD—maltodextrin, GA—gum arabic, WPC—whey protein concentrate

**Table 4:** Colour parameters L*, a*, b*, Chroma* and Hue angle of spray-dried nutritionally rich honey powders fortified with different carrier agents.

Samples	L*	a*	b*	Chroma*	Hue Angle (°)
Honey + MD	35.94 ± 0.18 ^c^	0.68 ± 0.03 ^c^	5.82 ± 0.1 ^d^	5.86 ± 0.10 ^c^	83.30 ± 0.28 ^a^
Honey + GA	34.32 ± 0.51 ^c^	1.17 ± 0.04 ^b^	8.36 ± 0.25 ^c^	8.44 ± 0.25 ^b^	82.03 ± 0.25 ^b^
Honey + WPC	30.74 ± 0.85 ^d^	1.89 ± 0.07 ^a^	9.52 ± 0.34 ^b^	9.52 ± 0.34 ^a^	78.76 ± 0.15 ^c^
Honey + MD + extract (aonla + basil)	45.78 ± 0.32 ^a^	0.85 ± 0.12 ^c^	7.93 ± 0.18 ^c^	7.97 ± 0.17 ^c^	83.86 ± 0.99 ^a^
Honey + GA + extract (aonla + basil)	42.94 ± 0.29 ^a^	1.52 ± 0.20 ^b^	9.91 ± 0.14 ^b^	10.02 ± 0.11 ^b^	81.23 ± 1.26 ^b^
Honey + WPC + extract (aonla + basil)	40.88 ± 0.14 ^b^	2.17 ± 0.15 ^a^	11.64 ± 0.06 ^a^	11.84 ± 0.06 ^a^	79.41 ± 0.71 ^c^

Results are expressed as mean values ± standard deviations. Means in a column with same superscripts are not significantly different (*p* < 0.05). MD—maltodextrin, GA—gum arabic, WPC—whey protein concentrate.

**Table 5:** Total phenolic content (TPC), vitamin C content, and antioxidant activity (AOA) of spray-dried honey powder fortified with different carriers as assessed by Duncan’s multiple range test.

Powder Combination	TPC (mg of GAE/100 g)	Vitamin C (mg/100 g)	AOA (%)
Honey + MD	42.72 ± 0.33 ^c^	Not detected	64.63 ± 0.51 ^c^
Honey + GA	45.81 ± 0.35 ^b^	Not detected	66.73 ± 0.77 ^b^
Honey + WPC	51.13 ± 0.80 ^a^	Not detected	72.08 ± 0.57 ^a^
Honey + MD + Extract (Aonla & Basil)	58.62 ± 0.62 ^c^	85.26 ± 0.29 ^c^	74.85 ± 0.17 ^c^
Honey + GA + Extract (Aonla & Basil)	60.99 ± 0.17 ^b^	87.23 ± 0.47 ^b^	78.15 ± 0.21 ^b^
Honey + WPC + Extract (Aonla & Basil)	63.22 ± 0.63 ^a^	94.87 ± 0.18 ^a^	82.73 ± 0.42 ^a^

Results are expressed as mean values ± standard deviations. Means in a column with same superscripts are not significantly different (*p* < 0.05). MD—maltodextrin, GA—gum arabic, WPC—whey protein concentrate, TPC—total phenolic content, AOA—antioxidant activity.
